# Prevalence of Hearing Loss and Hearing Aid Use Among Adults in France in the CONSTANCES Study

**DOI:** 10.1001/jamanetworkopen.2022.17633

**Published:** 2022-06-17

**Authors:** Quentin Lisan, Marcel Goldberg, Ghizlene Lahlou, Anna Ozguler, Sylvie Lemonnier, Xavier Jouven, Marie Zins, Jean-Philippe Empana

**Affiliations:** 1 Department of Head and Neck Surgery, Foch Hospital, Suresnes, France; 2 Université Paris Cité, INSERM, U970, Paris Cardiovascular Research Center, Integrative Epidemiology of Cardiovascular Disease, Paris, France; 3 Université Paris Cité, Population-based Cohorts Unit, INSERM, Paris Saclay University, Université de Versailles Saint-Quentin-en-Yvelines, UMS 011, Paris, France; 4 Assistance Publique–Hôpitaux de Paris Sorbonne Université, Groupe Hospitalo-Universitaire Pitié-Salpêtrière, Département d’Oto-Rhino-Laryngologie, Unité Fonctionnelle Implants Auditifs, Paris, France; 5 Institut de l’Audition/Institut Pasteur, Équipe Technologies and Gene Therapy for Deafness, Paris, France; 6 Department of Cardiology, European Hospital Georges Pompidou, Assistance Publique–Hôpitaux de Paris, Paris, France

## Abstract

**Importance:**

Although hearing loss is common in the population worldwide, the prevalence of hearing loss and hearing aid use is not known.

**Objective:**

To estimate the prevalence of hearing loss and hearing aid use in the adult French population.

**Design, Setting, and Participants:**

This cohort study used data from the CONSTANCES cohort, a representative sample of the French population. Volunteers aged 18 to 75 years were recruited at 21 preventive health centers between January 1, 2012, and December 31, 2019. The present study included participants with audiometric data.

**Main Outcomes and Measures:**

The main outcomes were prevalence of hearing loss and disabling hearing loss overall and by sex and age group and prevalence of self-reported hearing aid use among those with disabling hearing loss. Hearing loss was defined as a pure-tone average (PTA) in the better ear of 20 dB or higher, and disabling hearing loss was defined as a PTA in the better ear of 35 dB or higher.

**Results:**

Of 200 870 participants in the CONSTANCES study, 186 460 had full audiometric data and were included in this study (mean [SD] age, 47.1 [13.5] years); 100 330 (53.8%) were female, and 86 130 (46.2%) were male. Of these participants, 24.8% (95% CI, 24.6%-25.0%) had hearing loss and 4.3% (95% CI, 4.2%-4.4%) had disabling hearing loss. The prevalence rates of hearing loss increased from 3.4% (95% CI, 2.8%-3.9%) at age 18 to 25 years to 73.3% (95% CI, 69.5%-77.2%) at age 71 to 75 years among men and from 4.4% (95% CI, 3.9%-5.0%) at age 18 to 25 years to 64.1% (95% CI, 59.7%-68.4%) at age 71 to 75 years among women. The prevalence of disabling hearing loss increased from 0.3% (95% CI, 0.2%-0.4%) among participants aged 18 to 25 years to 23.3% (95% CI, 20.7%-26.0%) among participants aged 71 to 75 years. Among the 8050 participants with disabling hearing loss, 36.8% (95% CI, 35.8%-37.9%) reported using hearing aids, including 56.7% (95% CI, 38.9%-74.4%) aged 18 to 25 years and 32.9% (95% CI, 26.8%-39.2%) aged 71 to 75 years.

**Conclusions and Relevance:**

In this cohort study, hearing loss was prevalent in France, and the prevalence of hearing loss increased with age among both men and women. Hearing aids were underused, particularly among older individuals. These findings suggest that hearing loss prevention and screening in the French population are needed.

## Key Points

**Question:**

What is the prevalence of hearing loss and hearing aid use at the population level in France?

**Findings:**

In this cohort study including 186 460 French volunteers aged 18 to 75 years with audiometric data, 25% had hearing loss and 4% had disabling hearing loss. Among participants with disabling hearing loss, 37% reported using hearing aids.

**Meaning:**

The findings suggest that hearing loss is prevalent among French adults and that hearing aids are underused.

## Teaser

This cohort study estimates the prevalence of hearing loss and hearing aid use overall and by sex and age in the adult population in France.

## Introduction

Hearing loss affects 1.57 billion people worldwide, and projections suggest that 2.45 billion people will have hearing loss by 2050.^1^ Hearing loss is the third leading cause of years lived with disability and the first leading cause of years lived with disability among people older than 70 years. The World Health Organization recently estimated that unaddressed hearing loss represents an annual worldwide cost of $980 billion, including health care, education, productivity loss, and societal costs.^2,3^ In addition to constraining the ability to communicate, hearing loss is associated with delayed language among children, social isolation, altered quality of life, depression, cognitive decline, and dementia.^4,5,6,7,8^

Prevalence estimates of hearing loss are scarce, derived primarily from small and nonrepresentative studies, most often based on self-reports of hearing loss instead of objective audiometric testing, and often based on age-restricted cohorts.^1,9,10,11,12,13,14^ Existing studies collected data mostly in the 1990s,^15^ and the prevalence of hearing loss has evolved in parallel with the aging of the population.^16,17,18^ These limitations were highlighted in the 2019 Global Burden of Disease Study^1^ and in several other studies.^19,20,21^ Although some nationally representative studies have reported audiometric data on subsamples, such as the National Health and Nutrition Examination Survey,^22,23^ representative studies of large sample sizes are lacking in the field, notably in Europe. For instance, in France, the latest estimation of hearing loss prevalence was performed in 2008 and relied on self-reported answers, with estimates of hearing loss ranging from 8.5% to 16.1% depending on the question.^24^ Moreover, examining patient characteristics associated with hearing loss may help identify the populations at greatest risk of hearing loss and inform preventive strategies for hearing loss.

Although the association of hearing aids with improvements in several health-related outcomes are increasingly recognized,^14,25,26^ an underuse of hearing aids has been suggested.^27^ However, estimates of hearing aid use are not nationwide and were calculated mostly in older populations.^28,29^ This study used a large, nationwide representative sample of the French adult population with full data on audiometric testing to estimate the prevalence of hearing loss and hearing aid use in the French population and to assess the characteristics associated with hearing loss and hearing aid use.

## Methods

### Study Design

This cohort study included data from the CONSTANCES study, an ongoing nationwide study in France with a general purpose and a specific focus on occupational and social determinants of health and aging. The study design is detailed elsewhere.^30,31^ Between January 1, 2012, and December 31, 2019, adults aged 18 to 75 years were randomly selected from the National Health Insurance Fund (Caisse Nationale d’Assurance Maladie), which covers salaried workers who are professionally active or retired and their dependents (>85% of the French population), following a sampling scheme stratified by age, sex, socioeconomic status, and region of France to ensure a representative sample of the Caisse Nationale d’Assurance Maladie. Questionnaires on social, demographic, and health information, including personal and family history of diseases; events; and lifestyle behavior characteristics were self-administered at inclusion. A comprehensive health examination was performed at participating medical centers (21 centers across the French metropolitan territory) during which medical and clinical examinations, anthropometric measurements, and biological tests were performed. All included participants provided written informed consent. The CONSTANCES study followed the Declaration of Helsinki^47^ and was approved by the National Data Protection Authority and the institutional review board of the National Institute for Medical Research (INSERM). The present study was approved by the Comité d’Evaluation Ethique de l’Inserm. This study followed the Strengthening the Reporting of Observational Studies in Epidemiology (STROBE) reporting guideline.

### Hearing Loss and Hearing Aid Use

Participants were offered a hearing test at study inclusion, performed in a soundproof testing room (for 74.3% of participants) or an air-ambient quiet room depending on the recruitment center’s equipment. Air-conduction thresholds were determined for 0.5, 1, 2, 4, and 8 kHz, and testing was performed from −10 dB to 85 dB in 5-dB increments. All measuring processes were performed in accordance with the ISO 8253-1 standard.^48^ The pure-tone average (PTA) was calculated as the mean of the 0.5-, 1-, 2-, and 4-kHz frequencies for each ear. No hearing loss was defined as a PTA in the better ear of less than 20 dB, hearing loss as a PTA in the better ear of 20 dB or higher, and disabling hearing loss as a PTA in the better ear of 35 dB or higher, in accordance with recent recommendations.^1,2,32^

Audiometric testing was not performed for participants who used hearing aids at baseline. These participants were considered to have disabling hearing loss (PTA in the better ear of ≥35 dB) in the main analysis. This level of hearing loss generally corresponds with the threshold value for which hearing aids are advised and mostly accepted by individuals.^2,10,27^

### Covariates

Diabetes was defined as self-reported treatment for diabetes or fasting blood glucose level of 126.1 mg/dL or higher (to convert to micromoles per liter, multiply by 0.0555). Hypertension was defined as self-reported treated hypertension or a measured systolic blood pressure of 140 mm Hg or higher or a diastolic blood pressure of 90 mm Hg or higher at study inclusion. Depression was defined as self-reported treated depression or a Center for Epidemiologic Studies Depression Scale score of 19 or higher, according to the validated cutoff of the French version.^33^ Prevalent cardiovascular diseases (CVDs) were defined as a self-reported history of myocardial infarction, angina, stroke, or peripheral arterial disease. Noise exposure at work was self-reported on the basis of the following question: “Do you work (or have you worked) in an atmosphere where you needed to raise your voice to speak to someone located less than 2 meters from you?” Living alone and currently working (yes or no) were recorded. Smoking was categorized as never, former smoker, and current smoker. The degree of diploma was assessed using the 2011 International Standard Classification of Education (ISCED)^49^ and categorized as primary education, lower secondary education, upper secondary education, bachelor’s degree or equivalent education, and master’s or doctoral degrees (corresponding to levels 0-1, 2, 3-4, 5-6, and 7-8, respectively, from the 2011 ISCED). Monthly household income was recorded and categorized into quartiles of European household income^34^ as less than €1000, €1000 to €1500, €1500 to €2100, and more than €2100 per month (to convert Euros to US dollars, multiply by 1.05). Living area was dichotomized as rural or urban.

### Statistical Analysis

Prevalence rates of hearing loss and disabling hearing loss were calculated in the total population and by age group and sex, and 95% CIs were estimated. Characteristics associated with hearing loss and disabling hearing loss were examined using separate logistic regression models, and odds ratios (ORs) and 95% CIs were estimated. Candidate covariates were selected based on prior literature^23^ and included sex, age, body mass index (BMI), history of noise exposure at work, diabetes, hypertension, prevalent CVD, depression, smoking status, degree of diploma, household income, living alone, currently working, and type of living area (rural vs urban). The prevalence of hearing aid use among participants with disabling hearing loss was estimated. Logistic regression modeling was used to quantify associations between covariates and hearing aid use among participants with disabling hearing loss.

Several additional analyses were performed to assess the robustness of the findings. First, prevalence rates were re-estimated using weighting coefficients that were calculated in the CONSTANCES study for participants included in 2013, 2014, 2015, and 2016. The weighting coefficients took into account a nonparticipation correction factor based on the passive follow-up of a control cohort of nonparticipants, as described elsewhere.^35^ Second, analysis of characteristics associated with hearing loss was repeated using PTA as a continuous variable. Linear regression analysis was performed with a regression spline for age because of the nonlinear association between age and PTA. Third, prevalence estimates and characteristics associated with disabling hearing loss were rerun after reclassification of participants with hearing aids as presenting with any hearing loss (instead of disabling hearing loss) because hearing aids may be beneficial even for mild hearing loss (PTA, 20-34 dB).^26^ Fourth, multivariable regression models were rerun after imputing missing data using multiple imputation by chain equation.^36^ Fifth, the rates of hearing loss and hearing aid use calculated in this study were used to estimate the prevalence of hearing loss and hearing aid use in Europe, accounting for the age and sex population structure of the European population (2019 census; direct standardization).^37^ All analyses were performed using R, version 3.5.1 (R Project for Statistical Computing).

## Results

### Study Sample

Of 200 870 participants recruited in the CONSTANCES cohort, 14 410 had missing audiometric data (technical issues [n = 8466], refusal by the participants [n = 45], and unknown causes [n = 5899]). After exclusions, the study population consisted of 186 460 participants with a mean (SD) age of 47.1 (13.5) years; 100 330 (53.8%) were female, and 86 130 (46.2%) were male. Compared with included participants, excluded participants were more often living alone in urban areas, were currently working, had a higher-degree diploma, and more often had depression but were less often exposed to noise at work and less often had hypertension (eTable 1 in the Supplement).

### Prevalence of Hearing Loss in the CONSTANCES Cohort

**Figure 1.**
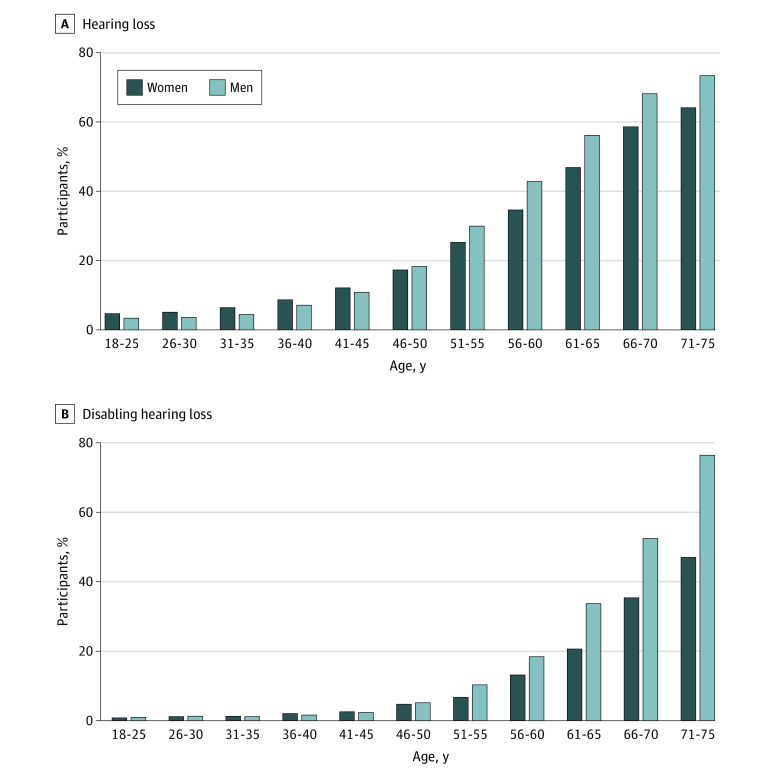
Prevalence of Hearing Loss and Disabling Hearing Loss by Age and Sex Among Participants From the CONSTANCES Study A total of 186 460 participants with full audiometric data from the CONSTANCES study were included in the present study. Hearing loss was defined as pure-tone average in the better ear of 20 dB or higher, and disabling hearing loss was defined as pure-tone average in the better ear of 35 dB or higher.

Overall, 24.8% (95% CI, 24.6%-25.0%) presented with any hearing loss. The prevalence of hearing loss increased with age, from 3.4% (95% CI, 2.8%-3.9%) at age 18 to 25 years to 73.3% (95% CI, 69.5%-77.2%) at age 71 to 75 years among men and from 4.4% (95% CI, 3.9%-5.0%) at age 18 to 25 years to 64.1% (95% CI, 59.7%-68.4%) at age 71 to 75 years among women (Figure 1 and eTable 2 in the Supplement). Prevalence rates were higher among women than among men up to age 41 to 45 years but lower among women after age 45 years. Among those aged 61 to 65 years, 51.4% (95% CI, 50.7%-52.0%) presented with any hearing loss (46.8% [95% CI, 45.9%-47.8%] of women and 56.1% [95% CI, 55.2%-57.1%] of men) (eTable 2 in the Supplement).

Disabling hearing loss was noted in 8050 study participants (4.3%; 95% CI, 4.2%-4.4%). Disabling hearing loss also increased with age, from 0.3% (95% CI, 0.2%-0.4%) among those aged 18 to 25 years to 23.3% (95% CI, 20.7%-26.0%) among participants aged 71 to 75 years (Figure 1 and eTable 2 in the Supplement). Differences in the prevalence of hearing loss by sex were found after age 51 years, and the prevalence was higher among men than among women.

### Characteristics Associated With Hearing Loss

The distribution of study participants’ characteristics according to hearing loss status is reported in Table 1. The mean (SD) PTA in the better ear was 10.4 (5.3) dB among participants without hearing loss, 26.4 (6.5) dB among participants with any hearing loss, and 40.2 (5.7) dB among participants with disabling hearing loss. The distribution of age, history of noise exposure at work, and cardiometabolic comorbidities increased with hearing loss severity, and the opposite results were seen for participants who were currently working, had a higher diploma, had a higher household income, were living alone, and were living in urban areas.

**Table 1.** Characteristics Associated With Hearing Loss and Disabling Hearing Loss in Participants From the CONSTANCES Study^a^

Characteristic	Participants^b^				Odds ratio (95% CI)^c^	
	No hearing loss (n = 140 243)	Any hearing loss (n = 46 217)	Disabling hearing loss (n = 8050)	Any hearing loss^d^		Disabling hearing loss^e^
Sex						
Female	77 451 (55.2)	22 879 (49.5)	3433 (42.7)	1 [Reference]		1 [Reference]
Male	62 792 (44.8)	23 338 (50.5)	4617 (57.3)	1.13 (1.10-1.16)		1.34 (1.26-1.42)
Age, mean (SD), y	43.7 (12.6)	57.5 (10.4)	61.2 (8.6)	1.10 (1.09-1.10)		1.11 (1.11-1.12)
BMI, mean (SD)	24.6 (4.4)	26.2 (4.7)	26.7 (4.8)	1.03 (1.02-1.03)		1.02 (1.01-1.03)
PTA, mean (SD), dB						
Left ear	12.0 (6.6)	28.9 (8.7)	43.2 (8.4)	NA		NA
Right ear	12.8 (6.6)	29.0 (8.4)	42.8 (7.8)	NA		NA
Better ear	10.4 (5.3)	26.4 (6.5)	40.2 (5.7)	NA		NA
Noise exposure at work	34 410 (25.4)	13 359 (29.9)	2778 (35.8)	1.24 (1.20-1.27)		1.49 (1.40-1.58)
Glucose level, mean (SD), mg/dL	95.5 (14.4)	100.9 (19.8)	102.7 (21.6)	NA		NA
Total cholesterol level, mean (SD), mg/dL	204.6 (42.5)	220.1 (42.5)	220.1 (42.5)	NA		NA
Blood pressure, mean (SD), mm Hg						
Systolic	125.5 (15.4)	133.6 (17.6)	136.7 (17.8)	NA		NA
Diastolic	75.3 (9.7)	78.5 (10.0)	79.2 (10.0)	NA		NA
Diabetes	3625 (2.6)	3528 (7.6)	848 (10.5)	1.18 (1.11-1.26)		1.19 (1.10-1.31)
Hypertension	26 062 (18.6)	17 533 (38.0)	3718 (46.2)	0.99 (0.96-1.02)		1.04 (0.98-1.10)
Prevalent CVD	1925 (1.4)	2161 (4.8)	554 (7.1)	1.20 (1.11-1.29)		1.21 (1.08-1.36)
Depression	40 479 (28.9)	14 395 (31.2)	2542 (31.7)	1.07 (1.03-1.10)		1.14 (1.07-1.21)
Living alone	37 264 (27.1)	10 891 (24.2)	1872 (23.9)	0.94 (0.90-0.98)		0.92 (0.86-1.00)
Smoking						
Never	65 951 (49.1)	19 225 (43.8)	3278 (42.9)	1 [Reference]		1 [Reference]
Former	42 463 (31.6)	18 138 (41.3)	3409 (44.6)	1.05 (1.02-1.08)		1.04 (0.98-1.11)
Current	26 013 (19.4)	6550 (14.9)	953 (12.5)	1.20 (1.16-1.25)		1.08 (0.99-1.18)
Currently working	106 474 (78.1)	21 150 (47.9)	2543 (33.4)	0.86 (0.83-0.89)		0.82 (0.76-0.88)
Diploma^f^						
Primary education	2944 (2.1)	2210 (4.9)	488 (6.2)	1 [Reference]		1 [Reference]
Lower secondary education	5636 (4.1)	4779 (10.6)	1037 (13.3)	0.76 (0.70-0.83)		0.75 (0.65-0.87)
Upper secondary	41 289 (30.0)	19 384 (43.1)	3597 (46.0)	0.74 (0.68-0.80)		0.71 (0.63-0.81)
Bachelor’s degree or equivalent	51 049 (37.1)	12 353 (27.5)	1772 (22.7)	0.55 (0.51-0.60)		0.54 (0.47-0.62)
Master’s or doctoral degree	36 577 (26.6)	6223 (13.8)	930 (11.9)	0.48 (0.44-0.52)		0.52 (0.45-0.61)
Household income, €/mo^g^				0.86 (0.83-0.89)		0.82 (0.76-0.88)
<1000	5450 (4.2)	1763 (4.2)	329 (4.5)	1 [Reference]		1 [Reference]
1000-1500	8807 (6.8)	3375 (8.0)	669 (9.2)	0.96 (0.88-1.05)		1.02 (0.86-1.21)
1500-2100	14 541 (11.2)	5463 (13.0)	1026 (14.2)	0.86 (0.79-0.93)		0.83 (0.71-0.98)
>2100	100 681 (77.8)	31 422 (74.8)	5222 (72.1)	0.75 (0.69-0.82)		0.75 (0.64-0.88)
Residential area						
Rural	26 402 (18.8)	10 267 (22.2)	1804 (22.4)	1 [Reference]		1 [Reference]
Urban	113 835 (81.2)	35 944 (77.8)	6247 (77.6)	0.90 (0.87-0.93)		0.97 (0.91-1.03)

Abbreviations: BMI, body mass index (calculated as weight in kilograms divided by height in meters squared); CVD, cardiovascular disease; PTA, pure-tone average.

SI conversion factors: To convert total cholesterol to micromoles per liter, multiply by 0.0259; and glucose to micromoles per liter, multiply by 0.0555.

^a^ A total of 186 460 participants with full audiometric data from the CONSTANCES study were included in the present study. Hearing loss was defined as PTA in the better ear of 20 dB or higher, and disabling hearing loss was defined as PTA in the better ear of 35 dB or higher.

^b^ Data are presented as number (percentage) of participants unless otherwise indicated.

^c^ Odds ratios and 95% CIs were obtained from multivariable logistic regression modeling (n = 150 413 participants; 36 047 participants were missing data on covariates).

^d^ Odds ratios measure the likelihood of having PTA of 20 dB or higher vs PTA less than 20 dB.

^e^ Odds ratios measure the likelihood of having PTA of 35 dB or higher vs PTA less than 35 dB.

^f^ Assessed using the 2011 International Standard Classification of Education.

^g^ To convert to US dollars, multiply by 1.05.

These results were similar in multivariate analysis, in which age, male sex, BMI, noise exposure at work, diabetes, prevalent CVD, depression, and smoking status were associated with higher odds of any hearing loss and disabling hearing loss. Higher educational level, higher household income, current working status, living in urban areas, and living alone were associated with lower odds of hearing loss and disabling hearing loss (Table 1).

### Prevalence of Hearing Aid Use

**Figure 2.**
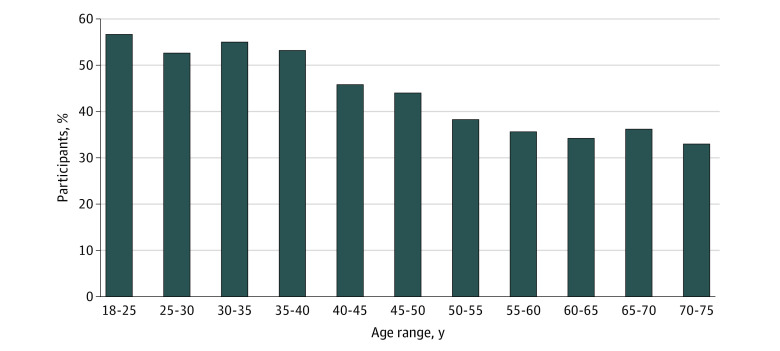
Use of Hearing Aids Among Participants With Disabling Hearing Loss From the CONSTANCES Cohort A total of 186 460 participants with full audiometric data from the CONSTANCES study were included in the present study. Disabling hearing loss was defined as pure-tone average in the better ear of 35 dB or higher.

Among 8050 participants with disabling hearing loss, 36.8% (95% CI, 35.8%-37.9%) reported using hearing aids. Hearing aid use decreased with age from 56.7% (95% CI, 38.9%-74.4%) among participants aged 18 to 25 years to 36.0% (95% CI, 34.3%-37.8%) among participants aged 65 to 70 years and 32.9% (95% CI, 26.8%-39.0%) among participants aged 71 to 75 years (Figure 2).

### Characteristics Associated With Hearing Aid Use

The characteristics of participants with and without hearing aids are described in Table 2. Multivariate analysis revealed that male sex, age, higher BMI, and current smoking status were associated with lower odds of hearing aid use, and a higher-degree diploma and household income were associated with higher odds of hearing aid use.

**Table 2.** Characteristics Associated With Hearing Aid Use Among Participants With Disabling Hearing Loss From the CONSTANCES Study^a^

Characteristic	Participants^b^		Odds ratio (95% CI)^c^
	Without hearing aids (n = 5085)	With hearing aids (n = 2965)	
Sex			
Female	2016 (39.6)	1419 (47.9)	1 [Reference]
Male	3069 (60.4)	1546 (52.1)	0.77 (0.68-0.86)
Age, mean (SD), y	61.6 (8.0)	60.4 (9.5)	0.98 (0.97-0.99)
BMI, mean (SD)	27.1 (4.8)	26.2 (4.6)	0.98 (0.97-0.99)
Noise exposure at work	1795 (36.6)	982 (34.4)	1.11 (0.98-1.24)
Diabetes	571 (11.2)	277 (9.3)	1.14 (0.94-1.37)
Hypertension	2444 (48.1)	1273 (42.9)	1.01 (0.90-1.13)
Prevalent CVD	389 (7.9)	164 (5.7)	0.88 (0.70-1.10)
Depression	1576 (31.1)	964 (32.6)	1.10 (0.98-1.24)
Living alone	1219 (24.6)	652 (22.6)	1.01 (0.87-1.18)
Smoking			
Never	1985 (41.4)	1273 (44.7)	1 [Reference]
Former	2135 (44.5)	1291 (45.4)	0.98 (0.87-1.10)
Current	673 (14.0)	281 (9.9)	0.69 (0.58-0.82)
Currently working	1525 (31.9)	1017 (36.1)	0.90 (0.78-1.04)
Diploma^d^			
Primary education	369 (7.5)	119 (4.1)	1 [Reference]
Lower secondary education	730 (14.8)	307 (10.7)	1.19 (0.88-1.61)
Upper secondary	2392 (48.4)	1204 (41.8)	1.31 (1.00-1.73)
Bachelor’s degree or equivalent	963 (19.5)	809 (28.1)	1.85 (1.39-2.48)
Master’s or doctoral degree	485 (9.8)	444 (15.4)	2.26 (1.67-3.08)
Household income, €/mo^e^			
<1000	255 (5.6)	74 (2.7)	1 [Reference]
1000-1500	484 (10.6)	185 (6.9)	1.31 (0.91-1.89)
1500-2100	698 (15.4)	327 (12.1)	1.49 (1.06-2.14)
>2100	3105 (68.4)	2115 (78.3)	1.97 (1.40-2.80)
Residential area			
Rural	1172 (23.1)	631 (21.3)	1 [Reference]
Urban	3913 (76.9)	2333 (78.7)	1.03 (0.91-1.18)

Abbreviations: BMI, body mass index (calculated as weight in kilograms divided by height in meters squared); CVD, cardiovascular disease; PTA, pure-tone average.

^a^ A total of 186 460 participants with full audiometric data from the CONSTANCES study were included in the present study. Disabling hearing loss was defined as PTA in the better ear of 35 dB or higher.

^b^ Data are presented as number (percentage) of participants unless otherwise indicated.

^c^ Odds ratios were obtained using multivariable logistic regression modeling, adjusted for covariates listed in the table. Odds ratios greater than 1 indicate a higher likelihood of using hearing aids, while odds ratios of 1 or less indicate a lower likelihood of using hearing aids.

^d^ Assessed using the 2011 International Standard Classification of Education.

^e^ To convert to US dollars, multiply by 1.05.

### Additional Analyses

Weighted and unweighted prevalence estimates of any hearing loss and disabling hearing loss were in the same range (eTable 3 in the Supplement). When examining hearing loss as a continuous outcome, results were similar to those of the main analysis except for sex, for which there was no association with hearing loss (eTable 4 in the Supplement). The level of hearing loss (PTA) according to age is shown in eFigure 1 in the Supplement. Reclassifying participants with hearing aids as having any hearing loss instead of having disabling hearing loss did not affect prevalence estimates and similar factors associated with hearing loss as in the main analysis (eTables 5-7 and eFigure 2 in the Supplement). Factors associated with hearing loss and hearing aid use remained unchanged after multiple imputations (eTable 8 in the Supplement). European estimates revealed 135.9 million people with any hearing loss and 26.7 million people with disabling hearing loss (eTable 9 in the Supplement).

## Discussion

In this cohort study of a nationally representative sample of the French adult population, 24.8% of participants had any hearing loss and 4.3% had disabling hearing loss; among those with disabling hearing loss, 36.8% reported using hearing aids. Prevalence estimates of hearing loss using objective testing are scarce. Worldwide estimates of hearing loss provided by the 2019 Global Burden of Disease Study relied on 113 data sources from 54 countries and considered all ages from birth to 100 years,^1^ whereas ages 18 to 75 years were considered in the present study. Important differences in hearing loss estimates were noted between the Global Burden of Disease Study and the current study. In the Global Burden of Disease Study, 11.1% of the French population was estimated to have hearing loss^1^ compared with more than twice that (24.8%) in the present study. However, only 1 data source is available for France in the Global Burden of Disease Study,^1^ and it reports newborn hearing screening.

To our knowledge, the Nord-Trøndelag Health Study (HUNT) study from Norway is the sole nationwide representative study in Europe with prevalence estimates of hearing loss using objective measures.^38^ Among 28 339 participants aged 19 to 100 years recruited in the last wave of the HUNT study (HUNT4), the prevalence of hearing loss was 2.7% at ages 20 to 44 years and 15.4% at ages 45 to 64 years compared with 7.2% at ages 20 to 44 years and 32.8% at ages 45 to 64 years in the present study. Similar discrepancies were observed for disabling hearing loss. These between-cohort disagreements may reflect different background population characteristics, but data on comorbidities are not yet available for HUNT4. Other community-based studies provided prevalence estimates of hearing loss in subsamples^22,23^ or in studies with incomplete audiometric testing^39,40^ or included selected age groups.^10,12^ Outside Europe, a South Korean study found that hearing loss was present in 13.4% of 18 650 participants aged 12 years or older.^41^ Data on comorbidities and hearing aid use were not reported in that study, however.

Hearing aids remain underused despite their association with improved outcomes, such as increased quality of life or reduced cognitive decline, compared with hearing loss without use of hearing aids.^14,25,26^ Orji et al^27^ estimated that 83% of individuals (all ages) worldwide and 77% of individuals in Europe with disabling hearing loss did not use hearing aids in an analysis derived from the 2019 Global Burden of Disease Study. A total of 63.2% of participants with disabling hearing loss in the present study did not use hearing aids. Of note, we observed a decrease in use of hearing aids associated with increasing age despite the prevalence of hearing loss increasing with age. Younger adults may have more social and professional interactions than older adults, which may be associated with greater use of hearing aids among younger adults than among older adults. Moreover, lower educational level and lower income were associated with underuse of hearing aids; thus, clinicians should pay attention to these populations. The underuse of hearing aids may be reconsidered as even greater than current estimates because indications for hearing aid use may expand beyond disabling hearing loss. Research suggests an association of hearing aids with improvement in mild hearing loss (PTA, 20-34 dB).^26^ Only 7% of individuals in the larger population of participants in the CONSTANCES cohort with any hearing loss were using hearing aids. Those findings are of particular importance in France, where full reimbursement for hearing aids was established after completion of this study.^42^ Thus, future estimates of hearing aid use in this cohort study may reveal the potential association of this public health policy with hearing aid adoption.

### Implications

Because of the association of hearing loss with health, the burden of hearing loss may have important public health implications. The first implication relies on the implementation of preventive strategies for hearing loss. Beyond early diagnosis and management of ear diseases, the current study identifies several factors associated with hearing loss that are modifiable or preventable, such as cardiometabolic risk factors (diabetes and prevalent CVD) and lifestyle risk factors (BMI and smoking status). Noise exposure at work was also associated with hearing loss; thus, noise prevention strategies at work may be beneficial. Socioeconomic inequalities among individuals with hearing loss are a challenge to address. The second implication relies on the screening and treatment of hearing loss. Although formal evidence is lacking regarding benefits associated with screening and treatment of hearing loss, as recently revealed by the US Preventive Task Force,^43,44^ policy makers may contemplate the implementation of screening programs based on the following considerations: (1) the heavy burden of hearing loss, particularly at 60 years or older, when more than half of the population experiences hearing loss; (2) the negative consequences of untreated hearing loss (eg, hearing loss is a modifiable risk factor for dementia)^4^; (3) the benefits associated with hearing rehabilitation^26^; and (4) the absence of adverse effects of screening for hearing loss.^43^ The third implication relies on implementation of strategies to improve hearing aid adoption among individuals with hearing loss. These programs should include strategies aimed at increasing awareness of and access to hearing aids, such as the strategies initiated in the US with the Over the Counter Hearing Aid Act in 2017^45^ or in France with better reimbursement for hearing aids since 2020.^42^

### Limitations

This study has limitations. First, participants with hearing aids at baseline did not have audiometric data. However, those participants were alternatively considered as presenting with disabling hearing loss or with any hearing loss and results remained similar. Second, bone conduction testing was not available, precluding assessment of the type of hearing loss (conductive, sensorineural, and mixed). Moreover, causes of hearing loss were unavailable, precluding estimations of the proportion of avoidable hearing loss cases due to ear diseases. Third, older individuals experience the highest prevalence of hearing loss, but the upper age limit in the current study was 75 years, which precluded the estimation of prevalence rates beyond this age. Fourth, as in all cohort studies including volunteers, a healthy participant bias cannot be ruled out; thus, the current prevalence rates may be underestimates. Fifth, the prevalence of hearing loss and hearing aid use in Europe estimated in the present study should be interpreted with caution because they represent estimates derived from a single country, whereas variation in the prevalence of hearing impairment across Europe has been reported.^46^

## Conclusions

In this cohort study, hearing loss was prevalent in France, and prevalence of hearing loss increased with age among both men and women. Hearing aids were underused, particularly among older individuals. Hearing loss is a field with unique opportunities because diagnosis and rehabilitation are accessible and mostly noninvasive.

## Notes

Supplement. **eTable 1.** Comparison of Excluded vs Included Participants **eTable 2.** Prevalence of Hearing Loss, Overall, by Sex and by Age Group in the CONSTANCES Cohort **eTable 3.** Unweighted and Weighted Prevalence Rates of Hearing Loss and Disabling Hearing Loss for 2013, 2014, 2015 and 2016 in CONSTANCES **eTable 4.** Determinants of Hearing Loss Examined as a Continuous Variable (Multivariable Linear Regression Analysis) **eTable 5.** Prevalence Estimates of Disabling Hearing Loss After Exclusion of Participants With Hearing Aids in CONSTANCES **eTable 6.** Characteristics Associated With Hearing Loss Severity After Reclassifying Participants With Hearing Aids Into the Group With Any Hearing Loss Instead of Disabling Hearing Loss **eTable 7.** Unweighted and Weighted Prevalence Rates of Disabling Hearing Loss for 2013, 2014, 2015 and 2016 in CONSTANCES After Exclusion of Participants With Hearing Aids **eTable 8.** Characteristics Associated With Hearing Loss and Hearing Aid Use After Multiple Imputations **eTable 9.** European Standardized Rates and Estimated Number of Individuals With Hearing Loss and Hearing Aids **eFigure 1.** Evolution of Pure Tone Average According to Age: Regression Spline, Adjusted for Covariates **eFigure 2.** Prevalence of Any Hearing Loss and Disabling Hearing Loss by Age and Sex After Reclassification of Participants With Hearing Aids Into the Group with Any Hearing Loss Instead of Disabling Hearing Loss
