# Error in Figure 1B

**DOI:** 10.1001/jamanetworkopen.2022.25053

**Published:** 2022-07-15

In the Original Investigation titled “Prevalence of Hearing Loss and Hearing Aid Use Among Adults in France in the CONSTANCES Study,”^1^ published June 17, 2022, in Figure 1B, the y-axis was incorrectly labeled from 0 to 80 instead of 0 to 30. This article has been corrected.^1^
