# Supplementary material for: Prevalence of Hearing Loss and Hearing Aid Use Among Adults in France in the CONSTANCES Study
Source: JAMA Netw Open. 2022 Jun 17;5(6):e2217633. doi: 10.1001/jamanetworkopen.2022.17633 (PMC9206187; doi:10.1001/jamanetworkopen.2022.17633)
Supplement: Supplement. — eTable 1. Comparison of Excluded vs Included Participants eTable 2. Prevalence of Hearing Loss, Overall, by Sex and by Age Group in the CONSTANCES Cohort eTable 3. Unweighted and Weighted Prevalence Rates of Hearing Loss and Disabling Hearing Loss for 2013, 2014, 2015 and 2016 in CONSTANCES eTable 4. Determinants of Hearing Loss Examined as a Continuous Variable (Multivariable Linear Regression Analysis) eTable 5. Prevalence Estimates of Disabling Hearing Loss After Exclusion of Participants With Hearing Aids in CONSTANCES eTable 6. Characteristics Associated With Hearing Loss Severity After Reclassifying Participants With Hearing Aids Into the Group With Any Hearing Loss Instead of Disabling Hearing Loss eTable 7. Unweighted and Weighted Prevalence Rates of Disabling Hearing Loss for 2013, 2014, 2015 and 2016 in CONSTANCES After Exclusion of Participants With Hearing Aids eTable 8. Characteristics Associated With Hearing Loss and Hearing Aid Use After Multiple Imputations eTable 9. European Standardized Rates and Estimated Number of Individuals With Hearing Loss and Hearing Aids eFigure 1. Evolution of Pure Tone Average According to Age: Regression Spline, Adjusted for Covariates eFigure 2. Prevalence of Any Hearing Loss and Disabling Hearing Loss by Age and Sex After Reclassification of Participants With Hearing Aids Into the Group with Any Hearing Loss Instead of Disabling Hearing Loss [file jamanetwopen-e2217633-s001.pdf]

Supplementary Online Content

Lisan Q, Goldberg M, Lahlou G, et al. Prevalence of hearing loss and hearing aid use among adults in France in the CONSTANCES study. *JAMA Netw Open.* 2022;5(6):e2217633. doi:10.1001/jamanetworkopen.2022.17633

eTable 1. Comparison of Excluded Versus Included Participants
eTable 2. Prevalence of Hearing Loss, Overall, by Sex and by Age Group in the CONSTANCES Cohort
eTable 3. Unweighted and Weighted Prevalence Rates of Hearing Loss and Disabling Hearing Loss for 2013, 2014, 2015 and 2016 in CONSTANCES
eTable 4. Determinants of Hearing Loss Examined as a Continuous Variable (Multivariable Linear Regression Analysis)
eTable 5. Prevalence Estimates of Disabling Hearing Loss After Exclusion of Participants With Hearing Aids in CONSTANCES
eTable 6. Characteristics Associated With Hearing Loss Severity After Reclassifying Participants With Hearing Aids Into the Group With Any Hearing Loss Instead of Disabling Hearing Loss
eTable 7. Unweighted and Weighted Prevalence Rates of Disabling Hearing Loss for 2013, 2014, 2015 and 2016 in CONSTANCES After Exclusion of Participants With Hearing Aids
eTable 8. Characteristics Associated With Hearing Loss and Hearing Aid Use After Multiple Imputations
eTable 9. European Standardized Rates and Estimated Number of Individuals With Hearing Loss and Hearing Aids
eFigure 1. Evolution of Pure Tone Average According to Age: Regression Spline, Adjusted for Covariates
eFigure 2. Prevalence of Any Hearing Loss and Disabling Hearing Loss by Age and Sex After Reclassification of Participants With Hearing Aids Into the Group with Any Hearing Loss Instead of Disabling Hearing Loss

This supplementary material has been provided by the authors to give readers additional information about their work.

eTable 1. Comparison of Excluded Versus Included Participants

	**Excluded N=14 410**	**Included N=186 460**
Male sex	6 818 (47.3)	86 130 (46.2)
Age (years)	46.2 ± 13.7	47.1 ± 13.5
BMI (kg/m^2)	24.4 ± 4.3	25.0 ± 4.5
Noise exposure at work	2 748 (19.8)	47 769 (26.5)
Glycemia (mmol/L)	5.24 ± 0.9	5.33 ± 0.9
Total cholesterol (mmol/L)	5.23 ± 1.0	5.40 ± 1.1
Systolic blood pressure (mmHg)	125.7 ± 16.1	127.5 ± 16.4
Diastolic blood pressure (mmHg)	75.6 ± 9.9	76.1 ± 9.9
Diabetes	512 (3.6)	7 153 (3.8)
Hypertension	2 670 (18.8)	43 595 (23.4)
Prevalent CVD	281 (2.0)	4 086 (2.3)
Depression	4 505 (31.4)	54 874 (29.5)
Living alone	4 838 (34.5)	48 155 (26.4)
Smoking		
Never	6 464 (47.6)	85 176 (47.8)
Former smoker	4 334 (31.9)	60 601 (34.0)
Current smoker	2 768 (20.4)	32 563 (18.3)
Currently working	10 031 (72.7)	127 624 (70.7)
Diploma (ISCED 2011)		
Primary education	415 (2.9)	5 154 (2.7)
Lower secondary education	642 (4.5)	10 415 (5.6)
Upper secondary	3 229 (22.6)	60 673 (32.7)
Bachelor or equivalent	4 592 (32.2)	63 402 (34.1)
Master and doctoral degree	5 135 (36.0)	42 800 (23.0)
Household income (€/month)		
<1000	695 (5.2)	7 213 (4.2)
1000-1500	940 (7.1)	12 182 (7.1)
1500-2100	1 514 (11.5)	20 004 (11.7)
>2100	10 016 (76.1)	132 103 (77.0)
Type of commune		
Rural	1 093 (7.59)	36 669 (19.7)
Urban	13 317 (92.4)	149 779 (80.3)

Abbreviations: BMI: body mass index; CVD: cardiovascular disease; ISCED: international standard classification of education.
Note: values are means ± SD or n (%).

© 2022 Lisan Q et al. *JAMA Network Open.*

eTable 2. Prevalence of Hearing Loss, Overall, by Sex and by Age Group in the CONSTANCES Cohort

	Hearing loss (PTA ≥20 dB) % (95% CI)	Disabling hearing loss (PTA ≥35 dB) % (95% CI)
**Overall prevalence**	24.8 (24.6 – 25.0)	4.3 (4.2 – 4.4)
**Both sexes combined**		
18-25 years	4.0 (3.6 – 4.4)	0.3 (0.2 – 0.4)
26-30 years	4.4 (4.1 – 4.8)	0.4 (0.3 – 0.5)
31-35 years	5.5 (5.2 – 5.9)	0.3 (0.3 – 0.4)
36-40 years	7.8 (7.5 – 8.2)	0.7 (0.6 – 0.8)
41-45 years	11.4 (11.0 – 11.8)	0.9 (0.8 – 1.1)
46-50 years	17.6 (17.1 – 18.1)	1.8 (1.6 – 2.0)
51-55 years	27.4 (26.8 – 28.0)	3.1 (2.8 – 3.3)
56-60 years	38.5 (37.8 – 39.1)	5.9 (5.5 – 6.2)
61-65 years	51.4 (50.7 – 52.0)	10.1 (9.7 – 10.5)
66-70 years	63.3 (62.6 – 64.0)	16.4 (15.9 – 16.9)
71-75 years	68.9 (66.0 – 71.8)	23.3 (20.7 – 26.0)
**Females**		
18-25 years	4.4 (3.9 – 5.0)	0.3 (0.2 – 0.5)
26-30 years	5.1 (4.6 – 5.6)	0.3 (0.2 – 0.5)
31-35 years	6.3 (5.8 – 6.8)	0.3 (0.2 – 0.4)
36-40 years	8.5 (8.0 – 9.0)	0.8 (0.6 – 0.9)
41-45 years	11.9 (11.4 – 12.5)	1.0 (0.8 – 1.1)
46-50 years	17.0 (16.3 – 17.7)	1.7 (1.5 – 2.0)
51-55 years	25.2 (24.4 – 26.1)	2.5 (2.2 – 2.8)
56-60 years	34.6 (33.7 – 35.5)	4.9 (4.5 – 5.3)
61-65 years	46.8 (45.9 – 47.8)	7.7 (7.2 – 8.2)
66-70 years	58.6 (57.6 – 60.0)	13.3 (12.6 – 14.0)
71-75 years	64.1 (59.7 – 68.4)	17.6 (14.2 – 21.1)
**Males**		
18-25 years	3.4 (2.8 – 3.9)	0.4 (0.2 – 0.6)
26-30 years	3.6 (3.1 – 4.0)	0.4 (0.3 – 0.6)
31-35 years	4.7 (4.2 – 5.1)	0.4 (0.2 – 0.5)
36-40 years	7.0 (6.5 – 7.5)	0.6 (0.5 – 0.8)
41-45 years	10.8 (10.2 – 11.4)	0.9 (0.7 – 1.0)
46-50 years	18.3 (17.5 – 19.1)	1.9 (1.6 – 2.1)
51-55 years	29.9 (29.0 – 30.9)	3.8 (3.4 – 4.2)
56-60 years	42.8 (41.8 – 43.8)	6.9 (6.4 – 7.4)
61-65 years	56.1 (55.2 – 57.1)	12.6 (12.0 – 13.3)
66-70 years	68.1 (67.2 – 69.1)	19.7 (18.9 – 20.5)
71-75 years	73.3 (69.5 – 77.2)	28.7 (24.7 – 32.6)

Abbreviations: PTA: pure tone average, in the better ear; CI: confidence interval; dB: decibel.

© 2022 Lisan Q et al. *JAMA Network Open.*

eTable 3. Unweighted and Weighted Prevalence Rates of Hearing Loss and Disabling Hearing Loss for 2013, 2014, 2015 and 2016 in CONSTANCES

	**Any hearing loss (PTA ≥20 dB) % (95% CI)**	**Disabling hearing loss (PTA ≥35 dB) % (95% CI)**
**Year**		
**2013**, *n=12 224*		
Unweighted	25.2 (24.4 – 26.0)	4.5 (4.2 – 4.9)
Weighted	21.5 (20.4 – 23.0)	4.1 (3.6 – 5.0)
**2014**, *n=17 713*		
Unweighted	26.6 (26.0 – 27.2)	4.4 (4.1 – 4.7)
Weighted	22.6 (21.7 – 23.0)	3.8 (3.4 – 4.0)
**2015**, *n=23 102*		
Unweighted	24.6 (24.1 – 25.2)	4.4 (4.1 – 4.6)
Weighted	24.3 (23.4 – 25.0)	4.9 (4.4 – 5.0)
**2016**, *n=22 062*		
Unweighted	25.3 (24.7 – 25.8)	4.4 (4.2 – 4.7)
Weighted	25.9 (25.0 – 27.0)	5.7 (5.2 – 6.0)

Abbreviations: PTA: pure tone average; dB: decibel; CI: confidence interval.

© 2022 Lisan Q et al. *JAMA Network Open.*

eTable 4. Determinants of Hearing Loss Examined as a Continuous Variable (Multivariable Linear Regression Analysis)

	Adjusted regression coefficient	95% CI
Male sex	-0.04	-0.12 – 0.04
BMI (per 1 kg/m^2 increase)	0.10	0.09 – 0.11
Noise exposure at work	0.68	0.60 – 0.77
Diabetes	0.98	0.77 – 1.19
Hypertension	-0.03	-0.13 – 0.07
Prevalent CVD	1.04	0.77 – 1.30
Depression	0.21	0.13 – 0.29
Living alone	-0.47	-0.57 – -0.37
Smoking		
Never	0	Reference
Former smoker	0.23	0.14 – 0.31
Current smoker	0.63	0.53 – 0.74
Diploma: ISCED 2011		
Level 0-1	0	Reference
Level 2	-1.19	-1.49 – -0.90
Level 3-4	-1.59	-1.85 – -1.34
Level 5-6	-2.65	-2.91 – -2.39
Level 7-8	-3.37	-3.63 – -3.10
Currently working	-0.15	-0.27 – -0.03
Type of commune		
Rural	0	Reference
Urban	-0.71	-0.81 – -0.61
Household income (€/month)		
<1000	0	Reference
1000-1500	-0.23	-0.48 – 0.02
1500-2100	-0.58	-0.81 – -0.34
>2100	-1.32	-1.54 – -1.09

Note: Results for age are given in the eFigure 1. Values are expressed as loss in dB, i.e., ß=0.68 for noise exposure at work means that noise exposure is associated with a 0.68 dB loss, whereas ß=-1.19 for ISCED level 2 indicates a mean difference of 1.19 dB in favor of ISCED level 2 compared to ISCED level 0-1 (reference). Regression coefficients are adjusted for the covariates listed in the table and age. Smoothing spline was used for age.
Abbreviations: CI: confidence interval, BMI: body mass index; CVD: cardiovascular disease; ISCED: international standard classification of education.

© 2022 Lisan Q et al. *JAMA Network Open.*

eTable 5. Prevalence Estimates of Disabling Hearing Loss After Exclusion of Participants With Hearing Aids in CONSTANCES

		**Disabling hearing loss (PTA in the better ear ≥35 dB)**
		% (95% CI)
**Overall**		2.8 (2.7 – 2.9)
**Overall – males**		3.6 (3.5 – 3.8)
**Overall - females**		2.0 (2.0 – 2.1)
**By age range**		
18-25 years		0.1 (0.1 – 0.2)
26-30 years		0.2 (0.1 – 0.2)
31-35 years		0.2 (0.1 – 0.2)
36-40 years		0.3 (0.2 – 0.4)
41-45 years		0.5 (0.4 – 0.6)
46-50 years		1.0 (0.9 – 1.1)
51-55 years		1.9 (1.7 – 2.1)
56-60 years		3.9 (3.6 – 4.1)
61-65 years		6.9 (6.5 – 7.2)
66-70 years		11.2 (10.7 – 11.6)
71-75 years		17.0 (14.5 – 19.4)

Abbreviations: PTA: pure tone average, in the better ear; CI: confidence interval; dB: decibel.

© 2022 Lisan Q et al. *JAMA Network Open.*

eTable 6. Characteristics Associated With Hearing Loss Severity After Reclassifying Participants With Hearing Aids Into the Group With Any Hearing Loss Instead of Disabling Hearing Loss

	**Study characteristics**			
	**Descriptive analysis**			**Multivariable logistic regression OR (95% CI)***
	**No hearing loss N=140 243 (75.2%)**	**Any hearing loss (PTA ≥20 dB) N=46 217 (24.8%)**	**Disabling hearing loss (PTA ≥35 dB) N=5 085 (2.7%)**	
Male sex	62 792 (44.8)	23 338 (50.5)	3 069 (60.4)	1.51 (1.40 – 1.63)
Age (years)	43.7 ± 12.6	57.5 ± 10.4	61.7 ± 8.0	1.12 (1.12 – 1.13)
BMI (kg/m^2)	24.6 ± 4.4	26.2 ± 4.7	27.0 ± 4.8	1.03 (1.02 – 1.03)
Audiometric data				
PTA left ear (dB)	12.0 ± 6.6	28.9 ± 8.7	43.3 ± 8.3	-
PTA right ear (dB)	12.8 ± 6.6	29.0 ± 8.4	42.8 ± 7.7	-
PTA better ear (dB)	10.4 ± 5.3	26.4 ± 6.5	40.2 ± 5.7	-
Noise exposure at work	34 410 (25.4)	13 359 (29.9)	1 795 (36.6)	1.46 (1.35 – 1.57)
Glycemia (mmol/L)	5.3 ± 0.8	5.6 ± 1.1	5.8 ± 1.2	-
Total cholesterol (mmol/L)	5.3 ± 1.1	5.7 ± 1.1	5.7 ± 1.1	-
Systolic blood pressure (mmHg)	125.5 ± 15.4	133.6 ± 17.6	137.6 ± 17.9	-
Diastolic blood pressure (mmHg)	75.3 ± 9.7	78.5 ± 10.0	79.6 ± 10.1	-
Diabetes	3 625 (2.6)	3 528 (7.6)	571 (11.3)	1.14 (1.02 – 1.28)
Hypertension	26 062(18.6)	17 533 (38.0)	2 444 (48.1)	1.03 (0.96 – 1.11)
Prevalent CVD	1925 (1.4)	2161 (4.8)	289 (7.9)	1.25 (1.10 – 1.43)
Depression	40 479 (28.9)	14 395 (31.2)	1 576 (31.1)	1.10 (1.02 – 1.19)
Living alone	37 264 (27.1)	10 891 (24.2)	1 219 (24.6)	0.91 (0.83 – 1.00)
Smoking				
Never	65 951 (49.1)	19 225 (43.8)	1 985 (41.4)	1.00 (ref)
Former smoker	42 463 (31.6)	18 138 (41.3)	2 135 (44.5)	1.06 (0.98 – 1.14)
Current smoker	26 013 (19.4)	6 550 (14.9)	673 (14.0)	1.26 (1.13 – 1.40)
Currently working	106 474 (78.1)	21 150 (47.9)	1 525 (31.9)	0.88 (0.80 – 0.96)
Diploma (ISCED 2011)				
Primary education	2 944 (2.1)	2 210 (4.9)	369 (7.5)	1.00 (ref)
Lower secondary education	5 636 (4.1)	4 779 (10.6)	730 (14.8)	0.73 (0.61 – 0.86)
Upper secondary	41 289 (30.0)	19 384 (43.1)	2 392 (48.4)	0.67 (0.58 – 0.78)
Bachelor or equivalent	51 049 (37.1)	12 353 (27.5)	963 (19.5)	0.43 (0.37 – 0.51)
Master’s and doctoral degree	36 577 (26.6)	6 223 (13.8)	485 (9.8)	0.39 (0.32 – 0.46)

© 2022 Lisan Q et al. *JAMA Network Open.*

eTable 6 (continued). Characteristics Associated With Hearing Loss Severity After Reclassifying Participants With Hearing Aids Into the Group With Any Hearing Loss Instead of Disabling Hearing Loss

	**Study characteristics**			
	**Descriptive analysis**			**Multivariable logistic regression OR (95% CI)***
	**No hearing loss N=140 243 (75.2%)**	**Any hearing loss (PTA ≥20 dB) N=46 217 (24.8%)**	**Disabling hearing loss (PTA ≥35 dB) N=5 085 (2.7%)**	
Household income (€/month)				
<1000	5 450 (4.2)	1 763 (4.2)	255 (5.6)	1.00 (ref)
1000-1500	8 807 (6.8)	3 375 (8.0)	484 (10.7)	0.94 (0.78 – 1.14)
1500-2100	14 541 (11.2)	5 463 (13.0)	698 (15.4)	0.74 (0.61 – 0.89)
>2100	100 681 (77.8)	31 422 (74.8)	3 106 (68.4)	0.60 (0.50 – 0.72)
Type of commune				
Rural	26 402 (18.8)	10 267 (22.2)	1 172 (23.0)	1.00 (ref)
Urban	113 835 (81.2)	35 944 (77.8)	3 913 (77.0)	0.96 (0.88 – 1.04)

Notes: values are mean ± SD or n (%).
Abbreviations: PTA: pure tone average; dB: decibel; CI: confidence interval; ref: reference category; BMI: body mass index; CVD: cardiovascular disease; ISCED: international standard classification of education.
* Odds ratios measure the likelihood of having PTA ≥35 dB vs. PTA <35 dB (N= 148,101; 38,359 participants with missing data)

© 2022 Lisan Q et al. *JAMA Network Open.*

eTable 7. Unweighted and Weighted Prevalence Rates of Disabling Hearing Loss for 2013, 2014, 2015 and 2016 in CONSTANCES After Exclusion of Participants With Hearing Aids

	**Disabling hearing loss (PTA ≥35 dB) % (95% CI)**
**Year**	
**2013**, *n=12 224*	
Unweighted	3.3 (2.9 – 3.6)
Weighted	3.2 (2.7 – 4.0)
**2014**, *n=17 713*	
Unweighted	2.9 (2.7 – 3.2)
Weighted	2.7 (2.3 – 3.0)
**2015**, *n=23 102*	
Unweighted	2.8 (2.6 – 3.0)
Weighted	3.3 (2.9 – 4.0)
**2016**, *n=22 062*	
Unweighted	2.8 (2.6 – 3.1)
Weighted	3.6 (3.2 – 4.0)

Abbreviations: HL: hearing loss; CI: confidence interval.

© 2022 Lisan Q et al. *JAMA Network Open.*

eTable 8. Characteristics Associated With Hearing Loss and Hearing Aid Use After Multiple Imputations

	Odds ratio (95% CI) for:			
	**Any hearing loss (PTA ≥20 dB)***	**Disabling hearing loss (PTA ≥ 35 dB)†**	**Disabling hearing loss (PTA ≥ 35 dB) after exclusion of hearing aids users†**	**Hearing aids use among disabling hearing loss**
Male sex	1.15 (1.12 – 1.18)	1.39 (1.32 – 1.46)	1.55 (1.45 – 1.65)	0.76 (0.68 – 0.84)
Age (per 1 year increase)	1.09 (1.09 – 1.10)	1.11 (1.11 – 1.11)	1.12 (1.11 – 1.12)	0.98 (0.97 – 0.99)
BMI (per 1 kg/m^2 increase)	1.02 (1.02 – 1.03)	1.02 (1.01 – 1.03)	1.03 (1.02 – 1.03)	0.98 (0.97 – 0.99)
Noise exposure at work	1.24 (1.21 – 1.28)	1.50 (1.43 – 1.58)	1.48 (1.40 – 1.58)	1.09 (0.98 – 1.21)
Living alone	0.95 (0.92 – 0.98)	0.95 (0.89 – 1.02)	0.93 (0.85 – 1.00)	1.05 (0.92 – 1.21)
Diabetes	1.18 (1.12 – 1.25)	1.18 (1.08 – 1.28)	1.16 (1.05 – 1.28)	1.10 (0.93 – 1.29)
Hypertension	1.00 (0.97 – 1.03)	1.05 (1.00 – 1.11)	1.05 (0.99 – 1.12)	0.99 (0.90 – 1.10)
Prevalent CVD	1.18 (1.10 – 1.27)	1.21 (1.10 – 1.33)	1.28 (1.15 – 1.44)	0.86 (0.71 – 1.05)
Depression	1.07 (1.04 – 1.10)	1.14 (1.08 – 1.20)	1.09 (1.02 – 1.17)	1.11 (1.00 – 1.24)
Smoking				
Never	1.00 (ref)	1.00 (ref)	1.00 (ref)	1.00 (ref)
Former smoker	1.04 (1.01 – 1.07)	1.02 (0.97 – 1.07)	1.01 (0.95 – 1.08)	1.00 (0.90 – 1.11)
Current smoker	1.19 (1.14 – 1.23)	1.06 (0.98 – 1.15)	1.21 (1.11 – 1.33)	0.67 (0.57 – 0.79)
Diploma (ISCED 2011)				
Primary education	1.00 (ref)	1.00 (ref)	1.00 (ref)	1.00 (ref)
Lower secondary education	0.76 (0.71 – 0.82)	0.75 (0.66 – 0.84)	0.72 (0.63 – 0.83)	1.21 (0.94 – 1.56)
Upper secondary	0.73 (0.69 – 0.78)	0.71 (0.64 – 0.79)	0.67 (0.58 – 0.75)	1.39 (1.11 – 1.76)
Bachelor or equivalent	0.55 (0.52 – 0.59)	0.54 (0.48 – 0.60)	0.44 (0.38 – 0.50)	1.98 (1.56 – 2.52)
Master and doctoral degree	0.47 (0.44 – 0.50)	0.50 (0.45 – 0.57)	0.39 (0.34 – 0.45)	2.26 (1.75 – 2.93)
Currently working	0.84 (0.81 – 0.86)	0.80 (0.75 – 0.86)	0.82 (0.76 – 0.90)	0.91 (0.80 – 1.02)
Type of commune				
Rural	1.00 (ref)	1.00 (ref)	1.00 (ref)	1.00 (ref)
Urban	0.91 (0.88 – 0.94)	0.97 (0.91 – 1.02)	0.96 (0.88 – 1.03)	1.03 (0.92 – 1.15)
Household income (€/month)				
<1000	1.00 (ref)	1.00 (ref)	1.00 (ref)	1.00 (ref)
1000-1500	0.92 (0.85 – 0.94)	0.94 (0.81 – 1.10)	0.87 (0.74 – 1.02)	1.33 (0.98 – 1.80)
1500-2100	0.82 (0.76 – 0.88)	0.82 (0.71 – 0.95)	0.74 (0.63 – 0.86)	1.55 (1.16 – 2.07)
>2100	0.73 (0.68 – 0.78)	0.74 (0.64 – 0.85)	0.60 (0.51 – 0.71)	2.04 (1.53 – 2.71)

* Odds ratios measure the likelihood of having PTA ≥20 dB vs. PTA <20 dB. † Odds ratios measure the likelihood of having PTA ≥35 dB vs. PTA <35 dB

Abbreviations: PTA: pure tone average; dB: decibel; CI: confidence interval; ref: reference category; BMI: body mass index; CVD: cardiovascular disease; ISCED: international standard classification of education.

Notes: Odds ratios were obtained using logistic regression modeling and adjusted for the covariates listed in the table.

© 2022 Lisan Q et al. *JAMA Network Open.*

eTable 9. European Standardized Rates and Estimated Number of Individuals With Hearing Loss and Hearing Aids

	Any hearing loss (PTA in the better ear ≥20 dB)			Disabling hearing loss (PTA in the better ear ≥35 dB)		
	% (95% CI)	Cases in millions (95% CI)		% (95% CI)	Cases in millions (95% CI)	% using hearing aids (95% CI)
**Overall prevalence**	26.0 (25.7 – 26.4)	135.9 (133.2 – 138.6)		5.1 (4.9 – 5.4)	26.7 (24.2 – 29.2)	35.9 (33.3 – 38.6)
**Overall prevalence – males**	26.4 (25.6 – 27.3)	67.6 (65.5 – 69.7)		5.8 (5.2 – 6.5)	14.9 (13.2 – 16.6)	31.6 (30.2 – 33.1)
**Overall prevalence – females**	25.4 (25.2 – 25.6)	68.3 (67.7 – 69.0)		4.4 (4.1 – 4.7)	11.8 (11.0 – 12.6)	42.4 (38.1 – 46.7)
**Prevalence by age group**						
18-25 years	3.9 (3.6 – 4.1)	1.5 (1.4 – 1.6)		0.3 (0.2 – 0.5)	0.1 (0.1 – 0.2)	54.5 (54.4 – 54.6)
26-30 years	4.3 (4.0 – 4.6)	2.0 (1.8 – 2.1)		0.4 (0.2 – 0.5)	0.2 (0.1 – 0.2)	51.6 (51.4 – 51.7)
31-35 years	5.4 (5.0 – 5.9)	2.9 (2.6 – 3.1)		0.3 (0.2 – 0.5)	0.2 (0.1 – 0.2)	56.4 (56.3 – 56.5)
36-40 years	7.8 (7.3 – 8.3)	4.1 (3.8 – 4.4)		0.7 (0.5 – 0.9)	0.4 (0.3 – 0.5)	53.2 (47.9 – 58.5)
41-45 years	11.4 (10.3 – 12.5)	5.9 (5.4 – 6.5)		0.9 (0.8 – 1.1)	0.5 (0.4 – 0.6)	45.8 (45.6 – 46.1)
46-50 years	17.6 (16.3 – 19.0)	9.2 (8.5 – 9.9)		1.8 (1.5 – 2.1)	0.9 (0.8 – 1.1)	43.7 (42.9 – 44.5)
51-55 years	27.5 (27.0 – 28.1)	14.2 (13.9 – 14.4)		3.1 (2.8 – 3.5)	1.6 (1.4 – 1.8)	38.0 (37.0 – 39.0)
56-60 years	38.5 (37.4 – 39.6)	20.3 (19.7 – 20.9)		5.9 (5.4 – 6.3)	3.1 (2.9 – 3.3)	35.6 (31.4 – 39.7)
61-65 years	51.1 (50.7 – 51.6)	25.0 (24.8 – 25.2)		10.0 (9.5 – 10.6)	4.9 (4.6 – 5.2)	34.5 (31.1 – 37.9)
66-70 years	62.9 (62.6 – 63.1)	27.0 (26.9 – 27.2)		16.1 (15.4 – 16.9)	6.9 (6.6 – 7.3)	36.3 (34.0 – 38.5)
71-75 years	68.1 (67.7 – 68.6)	23.9 (23.7 – 24.0)		22.5 (18.8 – 26.1)	7.9 (6.6 – 9.2)	34.4 (32.9 – 35.9)

Note: Rates are standardized to the European population census 2019; overall prevalence rates are age-standardized, and rates by age groups are sex-standardized.
Abbreviations: PTA: pure tone average; dB: decibel; CI: confidence interval.

© 2022 Lisan Q et al. *JAMA Network Open.*

eFigure 1. Evolution of Pure Tone Average According to Age: Regression Spline, Adjusted for Covariates

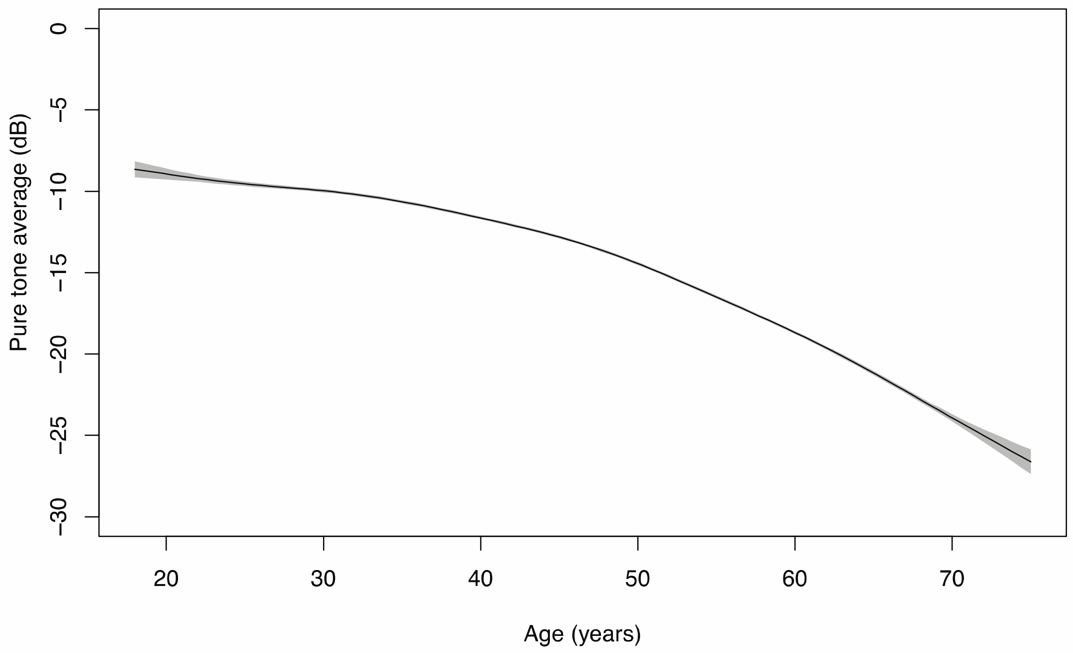

Note: Shaded area represents confidence interval. The multivariate regression model was adjusted for BMI, noise exposure at work, diabetes, hypertension, prevalent CVD, living alone, household income, depression, smoking status, level of diploma, currently working and type of commune.
Abbreviations: dB: decibel.

© 2022 Lisan Q et al. *JAMA Network Open.*

eFigure 2. Prevalence of Any Hearing Loss and Disabling Hearing Loss by Age and Sex After Reclassification of Participants With Hearing Aids Into the Group with Any Hearing Loss Instead of Disabling Hearing Loss

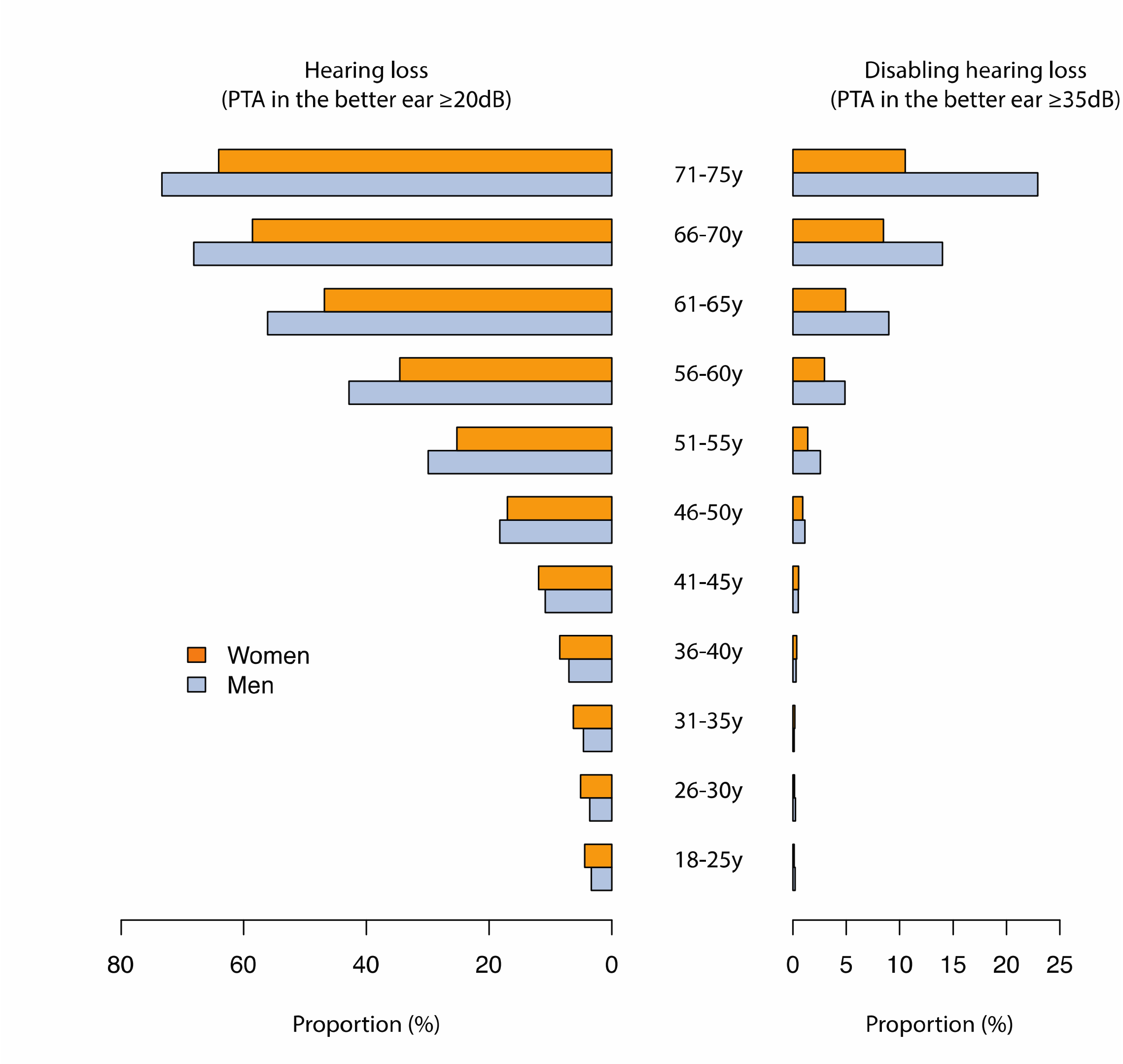

Abbreviations: PTA: pure tone average; dB: decibel.

© 2022 Lisan Q et al. *JAMA Network Open.*
